# Hepatocellular Carcinoma-propagating Cells are Detectable by Side Population Analysis and Possess an Expression Profile Reflective of a Primitive Origin

**DOI:** 10.1038/srep34856

**Published:** 2016-10-11

**Authors:** Honghai Xia, Jun Cao, Qing Li, Yang Lv, Weidong Jia, Weihua Ren, Qingyu Cheng, Xiaoyuan Song, Geliang Xu

**Affiliations:** 1 Graduate School, Tianjin Medical University, No. 22, Qixiangtai Road, Tianjin 300070, China; 2 Anhui Province Key Laboratory of Hepatopancreatobiliary Surgery, Anhui Provincial Hospital, No. 17, Lujiang Road, Hefei 230001, China; 3 CAS Key Laboratory of Brain Function and Disease, CAS Center for Excellence in Molecular Cell Science, Collaborative Innovation Center of Chemistry for Life Sciences, School of Life Sciences, University of Science and Technology of China, No. 443, Huangshan Road, Hefei 230001, China; 4 Central Laboratory of Medical research center, Anhui Provincial Hospital, No. 17, Lujiang Road, Hefei 230001, China; 5 Department of Hepatic Surgery, Anhui Provincial Hospital, No. 17, Lujiang Road, Hefei 230001, China.

## Abstract

The recent identification of “Side Population” (SP) cells in a number of unrelated human cancers has renewed interests in the hypothesis of cancer stem cells. Here we isolated SP cells from HepG2 cells and 18 of the 21 fresh hepatocellular carcinoma (HCC) tissue samples. These SP cells have higher abilities of forming spheroids, invasion and migration. Tumors could generate only from SP, not non-SP (NSP), cells in a low dose of subcutaneous injection to the NOD/SCID mice (5 × 10^2^ cells/mouse). The mRNA microarray analysis of the SP vs. NSP cells isolated from HepG2 cells revealed that the SP cells express higher levels of pluripotency- and stem cell-associated transcription factors including *Klf4, NF-Ya, SALL4* and *HMGA2*. Some of the known hepatobiliary progenitor/stem cell markers, such as *Sox9* was also up-regulated. RT-qPCR analysis of the gene expression between SP cells and NSP cells isolated from both HepG2 cells and HCC tissue samples showed that most of the tested mRNAs’ changes were in consistent with the microarray data, including the general progenitor/stem cells markers such as *Klf4, NF-Ya, SALL4* and *HMGA2*, which were up-regulated in SP cells. Our data indicates that HCC cancer stem cells exist in HepG2 and HCC fresh tissue samples and can be isolated by SP assay.

Hepatocellular carcinoma (HCC) is the second leading cause of cancer-related deaths in China. Hepatectomy has been considered as one of the curative treatments, but more than 40% of patients develop recurrences within one year after the operations^1^. The underlying mechanism of the recurrence with such a high incidence remains elusive, despite the fact that it is widely studied.

In traditional concept, every cell from tumors can act as a seed to form a new tumor independently. A recent idea about tumor biogenesis is cancer stem cells (CSCs). In the CSCs hypothesis, only a very small subpopulation of tumor cells—CSCs, can form new tumors, and they have some of the stemness properties which are nearly infinite self-renewal, extensive invasion and migration. With these properties, CSCs can resist hypoxia^2^, chemotherapies^3^, radiotherapies^4^, and immunization effects^5^. Different studies have revealed that many markers can be used to isolate hepatocellular cancer stem cells (HCSCs), such as CD90^6^,^7^, CD44^8^, CD133^9^, CD13^10^,^11^, EpCAM^12^. However, these markers are distinctive but not interrelated in different HCC cell lines.

Through adenosine triphosphate (ATP)-binding cassette (ABC) membrane transporters, the so-called “Side Population” (SP) cells can efflux the DNA-binding dye Hoechst33342 and can be detected by flow cytometry^13^,^14^. SP cells sorting was initially applied to isolate hematopoietic stem cells and then used to enrich stem cell compartments in diverse tissues^15^,^16^. Recently, it has been proved to be enriched with CSCs in many tumors, and the method was also performed in some HCC cell lines^17^,^18^,^19^,^20^,^21^,^22^,^23^,^24^. However, it is not well explored in HCC cell line HepG2 and fresh tissues from HCC patients. In this study, we aimed to investigate whether the SP cells in HCC display the properties of HCSCs, and whether they express different genes from Non-SP (NSP) cells.

## Results

### Isolation of SP cells in hepatocellular carcinoma cells

**Figure 1:**
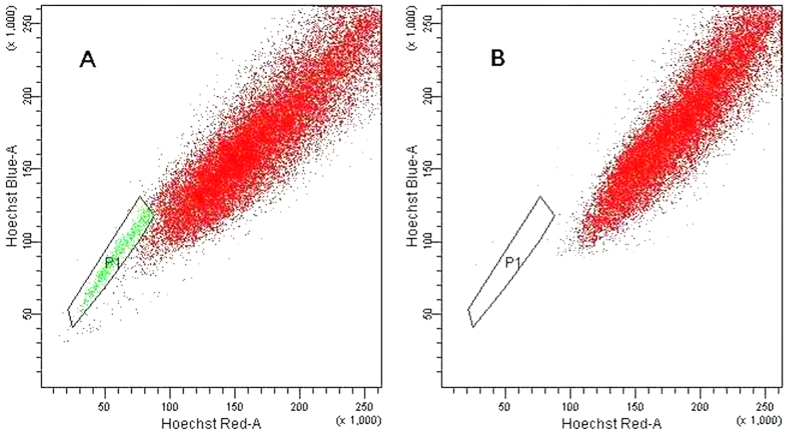
SP cells analysis. (**A)** SP cells were detected in HepG2 and 18 of the 21 fresh HCC tissue samples. The percentage of SP cells was 3.2% and 1.1–4.6%, respectively. Shown was one of the representative flow cytometry profiles, boxed green dots indicated the sorted SP cells. (**B**) SP cells (green dots) disappeared with verapamil (a calcium channel blocker) treatment. SP, Side Population.

SP cells were detected in HCC cell line HepG2 and 18 of the 21 HCC fresh tissue samples. The percentage was 3.2% and 1.1–4.6%, respectively (Fig. 1A). SP cells was disappeared when verapamil, a calcium channel blocker was added (Fig. 1B). SP and NSP cells were sorted separately for further investigations. Stained with trypan blue, it demonstrated that the average alive cells were 72.4%, and there was no difference between the two groups in morphology (data not shown).

### Cell proliferation abilities of the isolate SP cells

**Figure 2:**
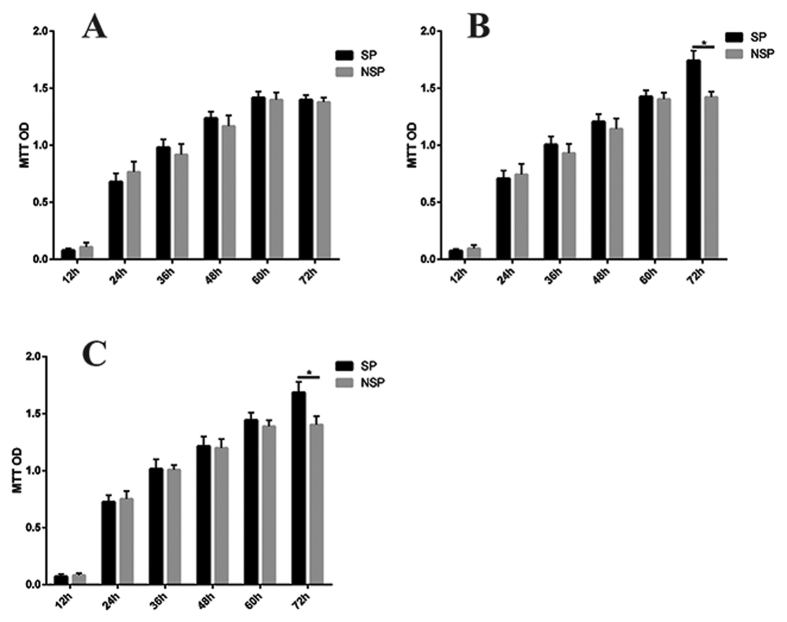
Cell proliferation arrays performed with MTT. (**A)** At 12 h, 24 h, 36 h, 48 h, 60 h and 72 h after sorting, there was no significant cell viability difference between SP and NSP cells in HepG2 cells and 16 fresh HCC tissue samples (*P* > 0.05). Shown was one representative figure. (**B,C)** At 72 h, the SP cells sorted from 2 fresh HCC tissue samples showed slight higher proliferation (*P < 0.05).

To investigate the *in vitro* proliferation abilities of SP and NSP cells, we performed MTT assy. At 12 h, 24 h, 36 h, 48 h, 60 h and 72 h after sorting, there was no significant (*P* > 0.05) cell viability difference between SP and NSP cells in HepG2 cells and in 16 fresh HCC tissue samples in medium supplemented with FBS (Fig. 2A). SP cells from the rest 2 fresh tissue samples showed slight higher proliferation at the 72 h time point after sorting (Fig. 2B,C).

### SP cells can form tumorspheres in serum-free medium

**Figure 3:**
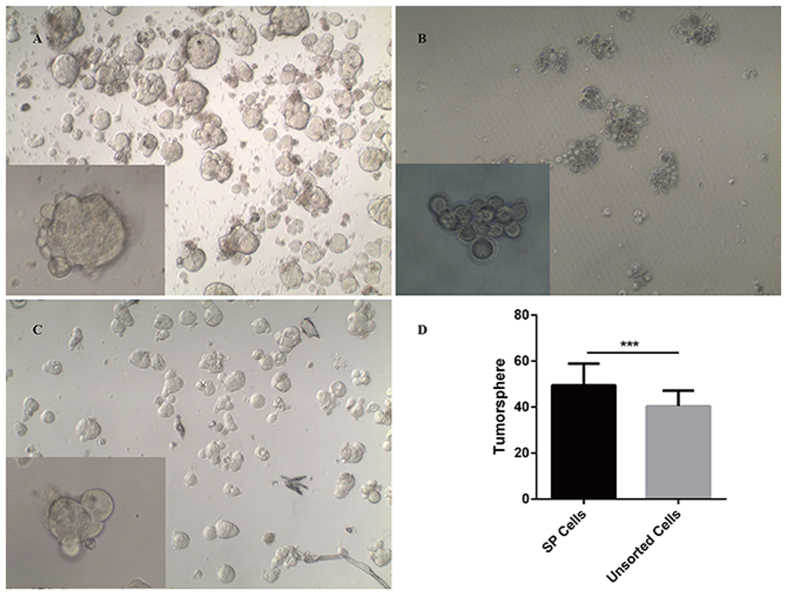
Tumorsphere cultures of SP, NSP and unsorted cells in serum-free medium. 9 days after seeded into 6-well ultra-low attachment plates in the serum-free medium, SP, NSP and unsorted cells (SP/NSP cells were sorted from freshly isolated HCC tissues) showed different abilities to form tumorspheres. (**A**) SP cells could form tumorspheres. (**B**) NSP cells could not form tumorspheres. Actually most of the NSP cells could not survive after such a long time serum-free medium culture, and the remaining cells were connected loosely and could be blowed into single cells easily. (**C)** Unsorted cells could form tumorspheres. (**D)** Tumorspheres from SP cells were significantly more than that from unsorted cells (49.48 ± 9.44 vs. 40.37 ± 6.48, F = 5.46, *P* < 0.05, n = 6).

It is well-known that most cells cannot survive and proliferate in serum-free medium, while stem cells can survive and form spheres, with some cell factors added to the serum-free medium. To investigate this property in different subpopulations of HCC tissue samples, we performed tumorsphere culture arrays on SP, NSP and unsorted cells of HCC fresh tissues. NSP cells could not form tumorsphere in DMEM/F12 supplemented with B27, EGF, bFGF (BD) and heparin. Actually most of the NSP cells could not survive after such a long time serum-free medium culture (9 days), and the remaining cells were connected loosely and could be blowed into single cells easily (Fig. 3B). However, SP cells and unsorted cells can form tumorspheres, and the tumorspheres from SP cells (Fig. 3A) were significantly more than that from unsorted cells (Fig. 3C,D; *P* < 0.05, n = 6).

### SP cells has more invasion and migration ability *in vitro*

**Figure 4:**
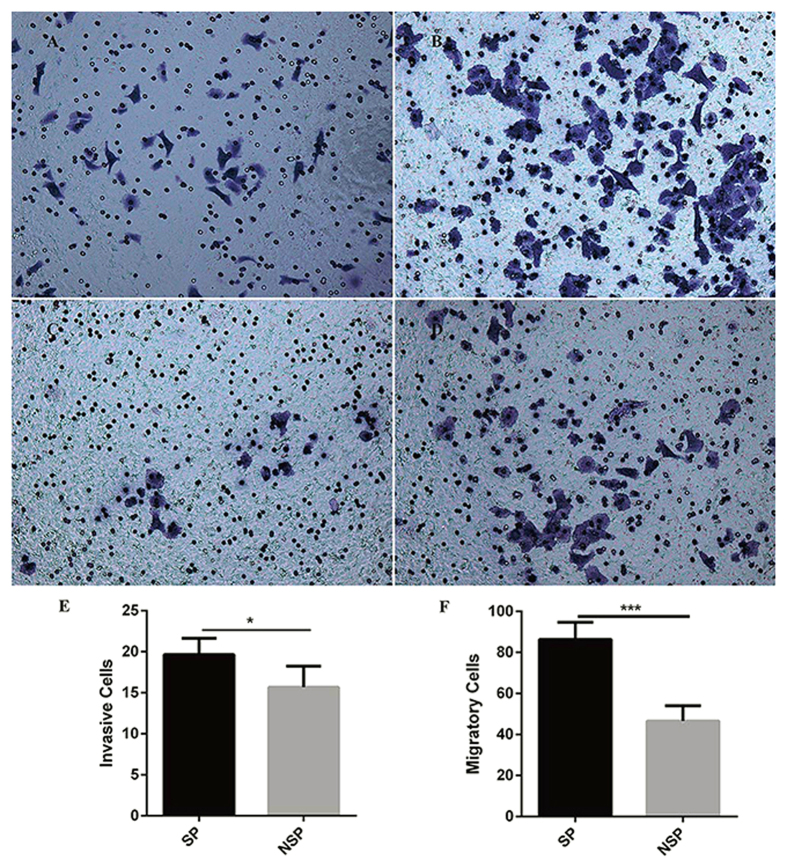
*In vitro* transwell cell invasion assay and *in vitro* transwell cell migration assay. SP and NSP cells sorted from freshly isolated HCC tissues were used for *in vitro* transwell cell invasion and cell migration assays. *In vitro* transwell cell invasion assay showed that SP cells (**A**) invaded more than NSP cells (**C**) with statistically significance (**E)** 19.67 ± 1.97 vs. 15.67 ± 2.58, F = 0.28, *P* < 0.05, n = 6). *In vitro* transwell cell migration assay showed that SP cells (**B**) migrated significantly more than NSP cells (**D**) with statistically significance (**F**) 86.33 ± 8.36 vs. 46.50 ± 7.58, F = 0.13, *P* < 0.05, n = 6).

To examine the invasion and migration ability of SP and NSP cells sorted from HCC fresh tissues, we performed *in vitro* invasion assay and migration assay with transwell Boyden chambers, different in adding or not extracellular matrix (ECM) gel to the chambers. *In vitro* transwell cell invasion assay showed that SP cells (Fig. 4A) invaded more than NSP cells (Fig. 4C) with statistically significance (Fig. 4E; 19.67 ± 1.97 vs. 15.67 ± 2.58, F = 0.28, *P* < 0.05, n = 6). *In vitro* transwell cell migration assay showed that SP cells (Fig. 4B) migrated significantly more than NSP cells (Fig. 4D) with statistically significance (Fig. 4F; 86.33 ± 8.36 vs. 46.50 ± 7.58, F = 0.13, *P* < 0.05, n = 6).

### SP cells produce tumors *in vivo* with low number of cells

**Figure 5:**
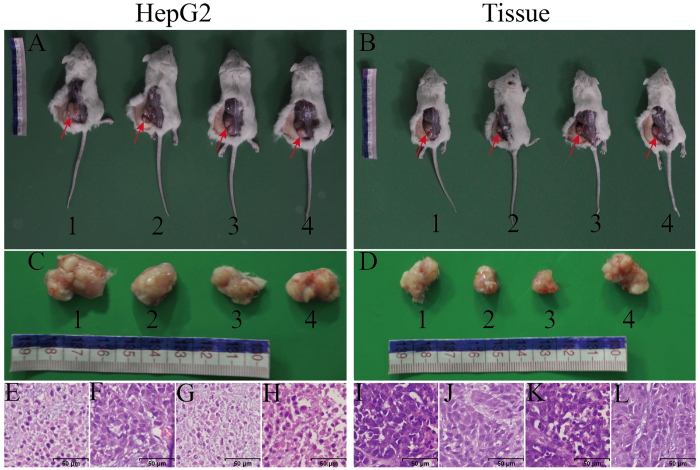
*In vivo* tumorigenicity of SP cells. Mice injected with SP cells (5 × 10^2^/mouse) (**A1–3**) and unsorted cells (1 × 10^6^/mouse) (**A4**) from HepG2 cells and tumors completely detached from the mice (**C**) were presented. The corresponding histological micrographs were shown in (**E–H)**. Representative picture of mice injected with SP cells (5 × 10^2^/mouse) (**B1–3**) and unsorted cells (1 × 10^6^/mouse) (**B4**) from 18 HCC patient tissues and tumors completely detached from the mice (**D**) were presented. The corresponding histological micrographs were shown in (**I–L**).

To examine the difference in the tumorigenicity assay on mice between SP and NSP cells, low number (5 × 10^2^/mouse) of SP cells from either HepG2 cells or HCC tissue samples were injected into NOD/SCID mice subcutaneously and tumor formation were examined ninety days after injection. In details, we prepared 19 groups of NOD/SCID mice with four mice in each group. In one group, three mice were injected with 500 SP cells (sorted from HepG2 cells) on the left back subcutaneous space, 500 NSP cells (sorted from HepG2 cells) on the right back subcutaneous space of the same three mice, and the remaining one mouse was injected with 1×10^6^ (large number) unsorted HepG2 cells as positive control. In parallel, the SP and NSP cells sorted from 18 HCC patients’ tissues were tested in the remaining 18 groups of NOD/SCID mice, following the HepG2 cells injection regimen. Ninety days after injection, we observed tumors in 52 of 57 mice injected with SP cells, whereas all the mice injected with NSP cells did not generate any tumor (The detailed information were shown in Supplementary Table S1). Thereinto, all of the 3 mice injected with SP cells from HepG2 cells (Fig. 5A1) and most of the mice injected with SP cells from patient HCC tissue samples generated tumors (representative pictures were shown in Fig. 5B1). Tumors also developed in all of the 19 positive control mice (Fig. 5A4). Histological analysis of low number of SP cells originated tumors showed similar features to those from large number of unsorted cells (Fig. 5E–L). The diameters of the tumor mass generated from SP cells and unsorted cells injection were 2.13 ± 0.44 cm and 2.20 ± 0.28 cm respectively (The detailed information was shown in Supplementary Table S1). There is also no significant difference between these two groups (*P* > 0.05, n = 71).

### SP cells express a primitive gene expression profile

**Figure 6:**
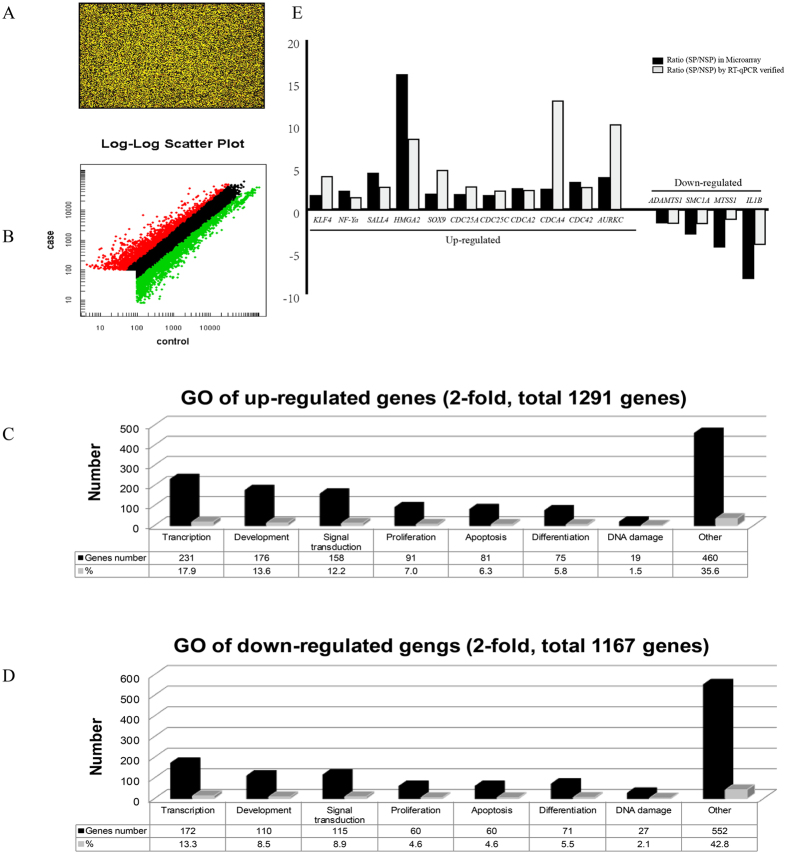
mRNA microarray analysis. (**A)** Human 8 × 60 k GeneChip image. (**B)** Scatter Plot SP vs. NSP cells from HepG2 cells. Red and green dots showed the up-regulated (ratio > 2) and down-regulated (ratio < 0.5) genes respectively in SP cells vs. NSP cells. (**C)** Gene Ontology-GO analysis of up-regulated genes in SP vs. NSP cells, shown were the biology process. (**D)** Gene Ontology-GO analysis of down-regulated genes in SP vs. NSP cells, shown were the biology process. (**E)** RT-qPCR assay validation of the differential expression of some genes. The microarray assay was performed on SP and NSP cells isolated form HepG2 cells, considering the rather huge individual differences in fresh HCC tissue samples.

**Figure 7:**
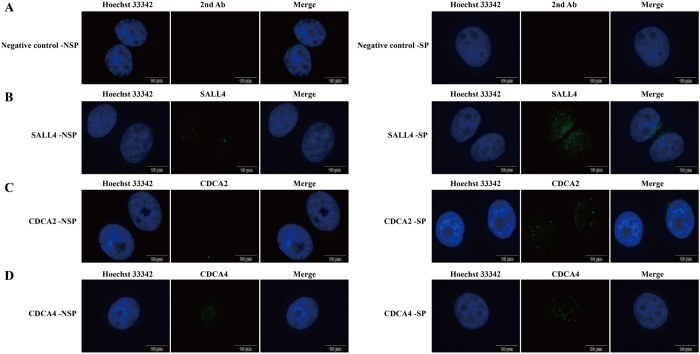
The relative protein expression level and spatio- subcellular localization of some of the genes in SP vs. NSP cells sorted from HepG2 cells. (**A)** The negative control. The cells were only incubated with Fluorescein isothiocyanate-conjugated secondary antibody before stained with Hoechst 33342 (blue). (**B–D)** Specific antibodies of anti-SALL4, CDCA2 and CDCA4 (Green) were used as primary antibody respectively. The Hoechst 33342 (blue) stained nuclei.

To systematically investigate the difference of gene expression and taking into account the individual differences between tissue samples, we further used mRNA microarray to analyze SP and NSP cells sorted from HepG2. The microarray data showed that 2057 genes’ expression were up-regulated (ratio > 2.0) and 3189 down-regulated (ratio < 0.5) in SP cells comparing to NSP cells (Fig. 6B). The genes were functionally categorized using CapitalBio Molecule Annotation System V3.0 (Bioinfo, Beijing, China). Through a statistical comparison of the significantly differentiated GO (gene ontology) annotation between SP and NSP cells, we observed that SP cells were more closely (both in gene number and in percentage) involved in transcription (GO: 0006355/0006350, 231 genes, 17.9%), development (176 genes, 13.6%) and signal transduction (GO: 0007165, 158 genes, 12.2%) (Fig. 6C). Meanwhile, cell adhesion (GO: 0007155) also holds an important position in SP cells (data not shown). Intriguingly, microarray indicated that pluripotency and stem cell-associated transcription factors including *Klf4, NF-Ya*^25^, *SALL4*^26^ and *HMGA2*^27^ were up-regulated in SP cells compared to NSP cells. Meanwhile, some of the known hepatobiliary progenitor/stem cell markers, such as *Sox9*^28^, and some of the key mediators of cell cycle progression such as *CDC25A* and *CDC25C* were also up-regulated. Whereas genes likely to be associated with development of cancer cachexia, such as *ADAMTS1*, and the key mediator of inflammatory response, such as *IL1B*, were down-regulated in SP cells (Table 1). We further verified some of the dys-regulated genes’ expression by RT-qPCR analysis between SP and NSP cells isolated from HepG2 cells (Table 1 and Fig. 6E), and also compared it with that from HCC tissue samples (data were shown in the Supplementary Table S2). The results showed that the general progenitor/stem cells markers such as *Klf4, NF-Ya, SALL4* and *HMGA2* were indeed up-regulated in SP cells from HCC tissue samples, the same as that from the HepG2 cells, and in consistent with the microarray data. In addition, immunofluorescence assay showed that the protein expression level changes between SP and NSP cells of some of the tested genes were also consistent with the microarray and RT-qPCR result, such as SALL4, CDCA2 and CDCA4 (Fig. 7). However, we did see some different mRNA expression between HCC tissue samples and HepG2 SP and NSP cells, such as *SOX9*. The reason for the difference between SP cells from HepG2 cells and HCC tissue samples could result from individual differences in patient tissue samples.

## Discussion

The nearly infinity capacity of proliferation, self-renew, and multipotential differentiation are the most characteristic properties of stem cells. By the hierarchical model of tumorgenesis, only subpopulation of the tumor cells that have some of the properties of stem cells, can generate tumor mass^29^,^30^. This subpopulation was named as CSCs, and was thought to be correlated with cancer recurrence and metastasis. In this study, HCC cell line HepG2 and fresh tissues of 21 HCC patients were sorted by flow cytometry to isolate SP cells and to estimate whether the SP cells show the properties of CSCs. SP cells were sorted and observed in HepG2 cells and 18 of the 21 HCC tissue samples. After sorting, we stained the cells with trypan blue and demonstrated more than 70% of the cells were alive. SP cells could then be used to further *in vitro* and *in vivo* investigations.

It has been reported that HCSCs can proliferate faster in cell line Huh7 and PLC/PRF/5, but it could be observed only at 72 h after plating and the ratio was not high^17^. In this study, we observed an almost identical capacity of proliferation in SP compared with NSP in the medium supplemented with FBS. In serum-free medium, we showed that SP cells could generate more tumorspheres than unsorted cells and NSP cells could not form tumorsphere. It is well-known that most CSCs are quiescent in cell cycle, as stem cells can divide symmetrically and asymmetrically^21^,^31^. The asymmetrical division indicates self-renew which can keep the quality and quantity of stem cells. Self-renew is supposed to be one of the reasons for CSCs to resist the harmful environment. Our study suggested that SP cells sorted from HepG2 and 18 of the 21 HCC tissue samples could not proliferate fast in total medium but could survive in medium without serum, indicating the property of CSCs, and thus the SP cells were enriched with HCSCs.

During the embryonic development, stem cells show higher migration ability and can migrate to distant organs with complex mechanisms^32^,^33^. It also has been suspected that tumor invasion and metastasis may be mediated by the CSCs subpopulation^34^. The migration and invasion ability of CSCs is higher than that of somatic cells. Our *in vitro* invasion and migration arrays clearly showed the disproportions between SP and NSP cells sorted from HepG2 and HCC tissues. Higher invasive and migratory phenotype of SP cells also revealed that they were enriched with CSCs. Through our research, we found that SP cells have some of the properties of CSCs: they can resist the adverse environment, they have more invasion and migration ability *in vitro* and produce tumor *in vivo* with low number of cells, but they do not show higher proliferation abilities compared to NSP cells.

Researches of tumor are currently placing emphasis on processes that are associated with developmental biology, specifically differentiation, embryonic signal transduction, and epigenetic regulation. According to the hierarchical model of cancer, carcinogenesis occurs when a stem cell acquires a mutation and initiates a stem cell-like counterpart^35^. Cancer cells heterogeneity is suspected to be related with CSCs’ proliferation, symmetrical or asymmetrical divisions, and differentiations^36^. Alpha-fetoprotein (AFP) expression in adults is often associated with hepatoma or teratoma. HCC cell line HepG2 is AFP positive, and different cells in HepG2 should equally express AFP at expectation. In our study, the expression of *AFP* in SP was 36.9 folds higher than that in NSP cells, suggesting that within the upstream, SP cells might lead to hierarchy of HCC cells.

In addition, our mRNA microarray analysis of HepG2 SP cells showed that they were enriched on MAPK, PPAR, Wnt, ErbB, VEGF signaling pathways, as well as focal adhesion and adherens junction, most of which are highly related to the properties of stem cells or CSCs. It has been reported that EGFR-mediated MAPK pathway reactivation leads to resistance to vemurafenib in BRAF-mutant colorectal cancers^37^. PAR-3-atypical protein kinase C can phosphorylate junctional adhesion molecule A to regulate cell-cell contact maturation, tight junctions^38^. Wnt is a signaling pathway made up of a complex network of proteins involved in embryogenesis, normal physiological processes and carcinogenesis^39^,^40^,^41^. RT-qPCR analysis of the gene expression between SP cells and NSP cells isolated from both HepG2 cells and HCC tissue samples showed that most of the tested mRNAs’ changes were in consistent with the microarray data. Undoubtedly, some RT-qPCR results did not consist with the microarray analysis, such as *c-MYC*, which is involved in cell proliferation and results in the formation of cancer. Previous studies have indicated that *c-MYC* plays a significant role in maintenance of glioma cancer stem cell and hematopoietic stem cell^42^,^43^. Although the microarray result showed the expression of *c-MYC* was down-regulated, our RT-qPCR analysis actually found that it was up-regulated in SP cells comparing to NSP cells, further demonstrating that SP cells in HCC display the properties of HCSCs. Moreover, as a transcriptional target of c-Myc, CDC25A showed up-regulated in SP cells through microarray analysis and RT-qPCR verification, which may contribute to cell cycle progression during differentiation^44^,^45^. We also found some different mRNA expression between HCC tissue samples and HepG2 SP and NSP cells, such as *SOX9*. The reason about the difference between SP cells from HepG2 cells and HCC tissues could result from individual differences in patient tissue samples. In our follow-up work, increasing our HCC patients’ sample in large number may increase the consistency and in the long run will be helpful to find some new therapy targets. In summary, SP assay might be a proper method to isolate CSCs in HCC, but the different gene expressions of SP cells should be further investigated in more cell lines and fresh tissue samples.

## Materials and Methods

### Cell Culture

The HCC cell line HepG2, obtained from Institute of Biochemistry and Cell Biology of the Chinese Academy of Sciences (Shanghai, China), was cultured in Dulbecco’s modified Eagle’s medium (DMEM; Hyclone, Logan, UT, USA) supplemented with 10% fetal bovine serum (FBS; Hyclone), 100 units/ml penicillin and 100 mg/ml streptomycin in a humidified incubator under 95% air and 5% CO_2_ at 37 °C. Cells were detached from the dishes with Trypsin-EDTA (Hyclone) and blowed into single-cell suspensions before flow cytometry.

### Tissue Samples

Primary HCC tissue samples were obtained from 21 patients undergoing surgical resection of primary HCC at Hepatic Surgical Department, Anhui Provincial Hospital, from January 2011 to July 2012. Sections were reviewed by two experienced pathologists to verify the histological assessment. All the specimens were hepatocellular cancer. Prior informed consent was obtained and all experimental protocols were approved by the Ethics Committees of the Anhui Provincial Hospital Clinical Research and University of Science and Technology of China. All experiments were performed in accordance with relevant guidelines and regulations, including any relevant details. Preparation of single-cell suspensions from HCC fresh specimens were performed in the C-tube of the gentle MACS Dissociator (Miltenyi Biotec, Cologne, Ger) added with HBSS, 3% FBS (Hyclone), and Trypsin-EDTA (Hyclone).

### Side Population Sorting using Flow Cytometry

Single-cells from cell line HepG2 and fresh HCC tissues were suspended at 1 × 10^6^/mL in DMEM medium (water bath at 37 °C beforehand). These cells were then incubated in water bath at 37 °C with 5 μg/mL Hoechst33342 (Sigma, StLouis, MO, USA) for 90 minutes, either alone or in the presence of 50 μg/mL verapamil (Sigma). After incubation, cells were centrifuged and suspended in HBSS at 4 °C. 1 μg/mL propidium iodide (BD, Pharmingen, SanDiego, CA) was added before the flow cytometry to distinguish the dead cells. Cell sorting was performed using an AriaII (BD) fluorescence activated cell sorting system (FACS). Hoechst33342 was excited with the UV laser at 375 nm and fluorescence emission was measured with 405/BP30 (Hoechst blue) and 570/BP20 (Hoechst red) optical filters. Propidium iodide was measured through the 630/BP30 filter.

### Cell Proliferation Assay

Cell proliferation assays were performed with 3-(4,5dimeth-ylthiazol-2-yl)-2, 5-diphenyl-2H-tetrazoliumbromide (MTT; Sigma). SP and NSP cells were seeded into a 96-well microplate at 1 × 10^3^ in 200 μl of medium supplemented with 10% FBS per well and maintained in a humidified incubator under 95% air and 5% CO_2_ at 37 °C. 24, 48 and 72 hours after sorting, 10 μl of MTT was added into triplicate wells and incubated at 37 °C for another 4 h, 100 μl of dimethylsulfoxide (DMSO; Sigma) was added and surged for 10 min. Then absorbance at 540 nm was measured to calculate the number of vital cells in each well by micro-enzyme-linked immunosorbent assay plate reader.

### Tumorsphere Culture

Suspended SP, NSP and unsorted cells were seeded into 6-well ultra-low attachment plates (Corning, Acton, MA, USA) at 1.5 × 10^3^ per well and cultured in serum-free DMEM/F12 (Hyclone) supplemented with B27 (Invitrogen, Gaithersburg, MD, USA), 20 ng/mL of EGF, 20 ng/mL of bFGF (BD) and 4 μg/mL of heparin (Sigma) as previously described^46^. After 9 days culture, wells were examined under an inverted microscope at ×200 magnification and the number of spheres in 9 fields of view was counted according to cell density per well.

### *In vitro* Transwell Cell Invasion Assay and *in vitro* Transwell Cell Migration Assay

The *in vitro* invasion capacity of SP and NSP cells was assayed using a 48-Transwell Boyden Chamber unit (Neuroprobe, Inc.) as previously described^47^. A Boyden chamber was separated into two compartments by a polycarbonate membrane with an 8-mm pore, over which a thin layer of ECM was dried. The ECM layer occluded the membrane pores and blocked the noninvasive cells from migrating. To the top chamber, 1.5 × 10^3^ SP or NSP cells in serum-free medium were added. In the lower chamber, DMEM with 10% FBS was added. After incubation at 37 °C in a 5% CO_2_ atmosphere for 48 hours, the noninvasive cells were removed with a cotton swab. The cells that had migrated through and adhered to the lower surface of the membrane were fixed with methanol for ten minutes and stained with crystal violet solution (0.1%). For quantification, the cells were counted using a microscope from five randomized fields at ×200 magnification. *In vitro* Transwell cell migration assay was performed using a protocol similar to that used for the invasive assay described above, however, ECM layer was not added to the chamber, and 3 × 10^3^ cells were added per chamber.

### Xenograft Transplantation

Non-obese Diabetic/Severe Combined Immunodeficiency mice (NOD/SCID) mice were purchased from Viatal River Laboratory Animal Technology Co. Ltd (Beijing, China). Prior informed consent was obtained and all experimental protocols were approved by the Ethics Committees of the Anhui Provincial Hospital Clinical Research and University of Science and Technology of China. All experiments were performed in accordance with relevant guidelines and regulations, including any relevant details. SP and NSP cells were suspended in 200 μl of DMEM and Matrigel (1:1) and respectively injected into the left and right back subcutaneous space of the male NOD/SCID mice (6 to 10 weeks old) under anesthesia (5 × 10^2^ cells/mouse/side). Tumor formation was observed weekly for 13 weeks. Subcutaneous tumors were fixed in formalin and embedded in paraffin. Sections were subjected to hematoxylin-eosin staining. These experiments were performed in accordance with the institutional guidelines for the use of laboratory animals.

### mRNA Microarray Analysis

Total RNA was extracted separately from SP and NSP cells using Trizol (Invitrogen) according to the manufacturer’s instructions. The RNA was further purified with NucleoSpin RNA clean-up (Macherey-Nagel, Ger). Microarray analysis was performed according to the standard protocol (Agilent Gene Chip Manual, Palo Alto, CA). First and second cDNA were generated from 1μg total RNA using T7 Oligo (dT) Primer, CbcScript kit, RNase H and DNA Polymerase (Invitrogen). The cRNA was then synthesized using the T7 Enzyme Mix (MN) and purified with the RNA Clean-up Kit (MN). Then cDNA was again generated from 5 μg cRNA using CbcScriptII, Random Prime and purified with PCR Nucleo Spin Extract II Kit (MN). The cDNA was labeled by Cy5-dCTP and Cy3-dCTP (GE Healthcare Cat. No. PA 55021/ PA53021) and then hybridized to human 8 × 60 k Gene Chip (Agilent). Array images were scanned using Agilent G2565CA Microarray Scanner and analyzed with Feature Extraction software. After experimental normalization, data mining including fold change measurement and categorization was conducted using GeneSpring system (Agilent).

### RT-qPCR

Total RNA were extracted separately from of SP and NSP cells using Eastep TM Universal RNA Extraction Kit (Promega) according to the manufacturer’s instructions. All qPCR reactions were performed in 10 ul volumes using AceQ qPCR SYBR Green Master Mix (Vazyme). Human GAPDH was used as reference gene.

### Statistical Analysis

All of the *in vitro* experiments were repeated at least three times. The data were expressed as means ± SD. Statistical analysis was performed by Student’s *t* test. The comparison of the incidence of tumor formation by *in vivo* mouse models was performed with Fisher’s exact test. All the analyses were performed using SPSS, version 10.0 (SPSS Inc, Chicago, Illinois, USA) and tests were two-sided with a significance level <0.05.

### Immunofluorescence

For immunofluorescence, SP and NSP cells cells were seeded onto sterile, acid–treated 12 mm glass coverslips in 24-well plates (NEST) separately. In general, 12–18 hours after adherence, cells were rinsed for 1 min with pre-warmed PHEM buffer (100 mM PIPES, 20 Mm HEPES, pH 6.9, 5 mM EGTA, 2 Mm MgCl2 and 4 M glycerol) and permeabilized for 1 min with PHEM plus 0.1% Triton X-100. Extracted cells were fixed with freshly prepared 3.7% paraformaldehyde in PHEM for 5 min at room temperature. After rinsing three times with PBS containing 0.05% Tween-20 (PBST), cells on the coverslips were blocked with 1% BSA (Sango) in PBST for 30 min. These cells were incubated with the various primary antibodies in a humidified chamber for 1 hour at room temperature or for 12 hours at 4 °C and then washed three times in PBST. Primary antibodies (Proteintech) were visualized using Fluorescein isothiocyanate-conjugated secondary antibody. DNA was stained with Hoechst 33342. Slides were examined under Olympus X71 Inverted fluorescence Microscope, and images were collected and analyzed with cellSens software.

### Ethics Committees Statement

Prior informed consent that the experiments on live vertebrates and/or higher invertebrates were obtained and all experimental protocols were approved by the Ethics Committees of the Anhui Provincial Hospital Clinical Research and University of Science and Technology of China. All experiments were performed in accordance with relevant guidelines and regulations, including any relevant details.

## Additional Information

**How to cite this article**: Xia, H. *et al*. Hepatocellular Carcinoma-propagating Cells are Detectable by Side Population Analysis and Possess an Expression Profile Reflective of a Primitive Origin. *Sci. Rep.* **6**, 34856; doi: 10.1038/srep34856 (2016).

## Supplementary Material

Supplementary Information

## Figures and Tables

**Table 1:** Representative Up- and Down- Regulated mRNAs in SP vs. NSP Cells from HepG2 Cells.

	Gene Symbol	Ratio (SP/NSP) in Microarray	Ratio (SP/NSP) by qPCR verified	Primary Accession
General progenitor/stem cells markers	*KLF4*	1.6253	3.869284223	NM_004235
	*NF-Ya*	2.1395	1.3771299	NM_002505
	*SALL4*	4.2563	2.602588977	NM_020436
	*HMGA2*	15.8719	8.264583042	NM_003483
Hepatobiliary progenitor/stem cell marker	*SOX9*	1.797	4.593347028	NM_000346
Key mediators of cell cycle progression	*CDC25A*	1.7425	2.655255395	NM_001789
	*CDC25C*	1.6276	2.160508414	NM_001790
Genes associated with cancer progression	*CDCA2*	2.4293	2.230677224	NM_152562
	*CDCA4*	2.3729	12.77967261	NM_017955
	*CDC42*	3.1785	2.575782137	NM_044472
Gene involved in oncogenic signal transduction	*AURKC*	3.7432	9.968123319	NM_001015878
Gene associated with development of cancer cachexia	*ADAMTS1*	0.6396	0.613877956	NM_006988
Gene related to DNA damage response	*SMC1A*	0.3474	0.604037634	NM_006306
Gene involved in signal transduction	*MTSS1*	0.2263	0.863955941	NM_014751
Key mediator of the inflammatory response	*IL1B*	0.123	0.243305895	NM_000576
