# Supplementary material for: Hepatocellular Carcinoma-propagating Cells are Detectable by Side Population Analysis and Possess an Expression Profile Reflective of a Primitive Origin
Source: Sci Rep. 2016 Oct 11;6:34856. doi: 10.1038/srep34856 (PMC5057076; doi:10.1038/srep34856)
Supplement: Supplementary Information [file srep34856-s1.pdf]

Hepatocellular Carcinoma-propagating Cells are Detectable by Side Population Analysis and Possess an Expression Profile Reflective of a Primitive Origin

Honghai Xia[a, b, #], Jun Cao[c, #], Qing Li[e], Yang Lv[b], Weidong Jia[b,d], Weihua Ren[b], Qingyu Cheng[c], Xiaoyuan Song[c] and Geliang Xu[a, b, d, *]

a. Graduate School, Tianjin Medical University, No. 22, Qixiangtai Road, Tianjin 300070, China

b. Anhui Province Key Laboratory of Hepatopancreatobiliary Surgery, Anhui Provincial Hospital, No. 17, Lujiang Road, Hefei 230001, China

c. CAS Key Laboratory of Brain Function and Disease, CAS Center for Excellence in Molecular Cell Science, Collaborative Innovation Center of Chemistry for Life Sciences, School of Life Sciences, University of Science and Technology of China, No.443, Huangshan Road, Hefei 230001, China

d. Department of Hepatic Surgery, Anhui Provincial Hospital

e. Central Laboratory of Medical research center, Anhui Provincial Hospital

[#] Equal contributors

*** Correspondence:** Geliang Xu, Anhui Province Key Laboratory of Hepatopancreatobiliary Surgery and Department of Hepatic Surgery, Anhui Provincial Hospital, No. 17, Lujiang Road, Hefei 230001, P.R. China. Tel: +86 551 62283916. Fax: +86 551 62282121

E-mail: 958304051@qq.com

Running title: Identification of HCSCs in HCC by SP Analysis

Supplementary Table S1 The Result in the Tumorigenicity Assay on NOD/SCID Mice between SP Cells, NSP Cells and Unsorted Cells from either HepG2 Cells or HCC Tissue Samples.

The diameters of the tumor mass from the SP cells and unsorted cells were 2.13±0.44cm and 2.20 ±0.28cm respectively. There is no significant different between these two groups (P＞0.05，n=71).

Group	Mice Number	SP Cells on the left back (Cell Number)	Tumor Mass (cm)	NSP Cells on the right back (Cell Number)	Tumor Mass (cm)	Unsorted Cells on the left back as positive control (Cell Number)	Tumor Mass (cm)
HepG2	1	500	2.8	500	0	0	
	2	500	2.5	500	0	0	
	3	500	1.9	500	0	0	
	4	0		0	0	1000000	2.6
Patient1	1	500	1.7	500	0	0	
	2	500	1.1	500	0	0	
	3	500	0	500	0	0	
	4	0		0	0	1000000	1.9
Patient2	1	500	2.2	500	0	0	
	2	500	2.6	500	0	0	
	3	500	2.1	500	0	0	
	4	0		0	0	1000000	2.8
Patient3	1	500	1.7	500	0	0	
	2	500	3.1	500	0	0	
	3	500	1.9	500	0	0	
	4	0		0	0	1000000	2
Patient4	1	500	2.3	500	0	0	
	2	500	1.8	500	0	0	
	3	500	0	500	0	0	
	4	0		0	0	1000000	2.5
Patient5	1	500	2.2	500	0	0	
	2	500	1.9	500	0	0	
	3	500	2	500	0	0	
	4	0		0	0	1000000	2.3
Patient6	1	500	1.8	500	0	0	
	2	500	0	500	0	0	
	3	500	2.1	500	0	0	
	4	0		0	0	1000000	2.2
Patient7	1	500	2.2	500	0	0	
	2	500	2	500	0	0	
	3	500	1.5	500	0	0	
	4	0		0	0	1000000	1.9
Patient8	1	500	2.8	500	0	0	
	2	500	2	500	0	0	
	3	500	2.2	500	0	0	
	4	0		0	0	1000000	2.1
Patient9	1	500	2.6	500	0	0	
	2	500	2.9	500	0	0	
	3	500	2.1	500	0	0	

	4	0		0	0	1000000	2.2
Patient10	1	500	2	500	0	0	
	2	500	2.5	500	0	0	
	3	500	0	500	0	0	
	4	0		0	0	1000000	1.9
Patient11	1	500	2.4	500	0	0	
	2	500	2.2	500	0	0	
	3	500	1.8	500	0	0	
	4	0		0	0	1000000	2.7
Patient12	1	500	1.7	500	0	0	
	2	500	2.1	500	0	0	
	3	500	2.2	500	0	0	
	4	0		0	0	1000000	2
Patient13	1	500	2.5	500	0	0	
	2	500	2.8	500	0	0	
	3	500	2.3	500	0	0	
	4	0		0	0	1000000	2.4
Patient14	1	500	2.1	500	0	0	
	2	500	1.2	500	0	0	
	3	500	1	500	0	0	
	4	0		0	0	1000000	2.2
Patient15	1	500	2.4	500	0	0	
	2	500	2.7	500	0	0	
	3	500	0	500	0	0	
	4	0		0	0	1000000	2.2
Patient16	1	500	2.6	500	0	0	
	2	500	2.2	500	0	0	
	3	500	1.8	500	0	0	
	4	0		0	0	1000000	1.9
Patient17	1	500	2.4	500	0	0	
	2	500	1.8	500	0	0	
	3	500	2.1	500	0	0	
	4	0		0	0	1000000	2
Patient18	1	500	2.1	500	0	0	
	2	500	1.8	500	0	0	
	3	500	2.3	500	0	0	
	4	0		0	0	1000000	2

Supplementary Table S2 Representative Up- and Down- Regulated mRNAs in SP vs NSP Cells from Fresh HCC Tissue Samples.

Examples of Up-Regulated genes in SP vs NSP Cells	Gene Symbol	Ratio (SP/NSP) in Microarray	Ratio (SP/NSP) by qPCR verified	Primary Accession
General progenitor/stem cells markers	*KLF4*	1.6253	1.114532815	NM_004235
	NF-Ya	2.1395	1.556309978	NM_002505
	SALL4	4.2563	9.263384103	NM_020436
	HMGA2	15.8719	10.94290953	NM_003483
Key mediator of cell cycle progression	*CDC25C*	1.6276	1.645846984	NM_001790
Hepatobiliary progenitor/stem cell marker	*SOX9*	1.797	0.243628838	NM_000346

Examples of Down-Regulated genes in SP vs NSP Cells	Gene Symbol	Ratio (SP/NSP) in Microarray	Ratio (SP/NSP) by qPCR verified	Primary Accession
Gene associated with development of cancer cachexia	*ADAMTS1*	0.6396	0.7847543	NM_006988
Key mediator of the inflammatory response	*IL1B*	0.123	0.065773745	NM_000576
